# Lotusin A: a novel pyrrole terpenoid hydrid from lotus roots (*Nelumbo nucifera* Gaertn.)

**DOI:** 10.1039/d5ra05354d

**Published:** 2025-12-15

**Authors:** Le Viet Ha Tran, Huu Canh Vo, To Hoang Long, Vinh Han La, Quoc Tuan Le, Minh Canh Nguyen, Duc Trung Le, Tran Dang Linh Nguyen, Thanh-Tung Phan, Ngo Thi Thuy Duong, Quang Ton That, Linh Tran, Minh-Tri Le, Khac-Minh Thai, Le-Thuy-Thuy-Trang Hoang, Huynh Nguyen Khanh Tran

**Affiliations:** a Faculty of Traditional Medicine, University of Medicine and Pharmacy at Ho Chi Minh City Vietnam; b Faculty of Chemistry, University of Science Ho Chi Minh City 70000 Vietnam; c Faculty of Pharmacy, University of Health Sciences Ho Chi Minh City Vietnam thnkhanh@uhsvnu.edu.vn +84939775593; d Research Center for Discovery and Development of Healthcare Products, Vietnam National University Ho Chi Minh City Ho Chi Minh City Vietnam; e Vietnam National University Ho Chi Minh City 70000 Vietnam; f Laboratory of Advanced Materials Chemistry, Institute for Advanced Study in Technology, Ton Duc Thang University Ho Chi Minh City Vietnam; g Faculty of Applied Sciences, Ton Duc Thang University Ho Chi Minh City Vietnam

## Abstract

*Nelumbo nucifera* Gaertn. has been used as a traditional medicine and food. To date, many unknown constituents have been discovered. Most of the researches on secondary metabolites and their pharmacological properties focus on alkaloid derivatives. In this study, a novel pyrrole terpenoid hydrid, lotusin A (1), and five reported substances, cholestanol (2), stigmast-4-en-3-one (3), quercetin (4), isorhamnetin (5), and norartocarpetin (6), were isolated from the methanol extract of the roots of *N. nucifera* Gaertn. The structures (1–6) were identified *via* spectroscopic analyses of NMR (1D and 2D), HR-ESIMS, and comparisons with those previously reported in the literature. Compound 1, a pyrrole terpenoid hydrid, is a unique isolate from plant natural products. Compounds 1 and 2 exhibited inhibitory activity against NO production, while all compounds showed growth inhibition of six bacterial strains. Results indicated that 1 moderately inhibited NO production with an IC_50_ value of 21.5 µM, but no inhibition of microbial growth was observed at a concentration of 10 µM. Besides, compound 1 exhibited good inhibition of α-glucosidase (IC_50_ = 18.2 µM); in contrast, this result was not observed for other compounds. From the result of molecular docking data, 1 has good interaction to with both of pro-inflammatory cytokines (iNOS, COX-2, TNF-α, IL-1β, and IL-6); transcription factors (Nrf2 and NF-κB), diabetes enzyme with binding energies of −6.4, −6.4, −5.0, −5.8, −6.1 kcal mol^−1^; −6.1, −6.6 kcal mol; and −6.4 kcal mol^−1^, respectively, resulted that 1 is the promising candidate for anti-inflammatory and α-glucosidase inhibition. Moreover, *in silico* ADMET and toxicity predictions indicated that 1 had favourable safety and pharmacokinetic profiles.

## Abstract 2

*Nelumbo nucifera* Gaertn. has been used as a traditional medicine and food. To date, many unknown constituents have been discovered.

## Graphical abstract

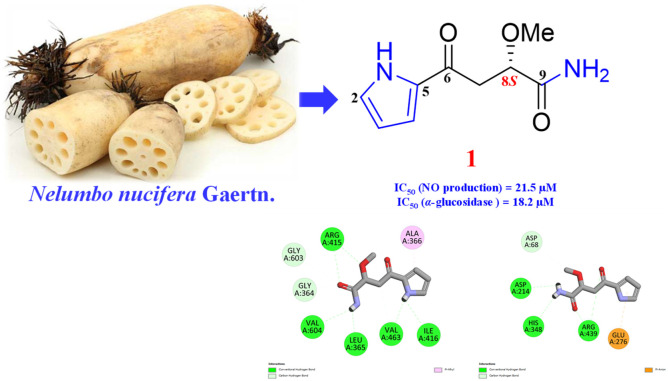

## Introduction

Lotus is a perennial aquatic plant belonging to the Nelumbonaceae family, with one *Nelumbo* genus having two species, namely, *Nelumbo nucifera* Gaertn. and *Nelumbo lutea* Pear., which are commonly planted in Asian countries.^1^ All parts of *N. nucifera* are highly beneficial as a traditional medicine for the treatment of pharyngopathy, pectoralgia, spermatorrhoea, leucoderma, smallpox, dysentery, cough, haematemesis, epistaxis, haemoptysis, haematuria, metrorrhagia, hyperlipidaemia, fever, cholera, hepatopathy, and hyperdipsia. Active constituents are responsible for these uses and can be isolated and identified as alkaloids, steroids, triterpenoids, flavonoids, glycosides, and polyphenols. Extracts from the leaf, rhizome, seed and flower display a variety of activities: anti-ischemic, antioxidant, anticancer, antiviral, anti-obesity, lipolytic, hypocholesterolaemic, antipyretic, hepatoprotective, hypoglycaemic, antidiarrhoeal, antifungal, antibacterial, anti-inflammatory, and diuretic activities.^2^ Nuciferine, a major active component in *N. nucifera*, could prevent breast cancer cell-mediated bone destruction through the inhibition of the growth of MDA-MB-231 and MCF-7 human breast cancer cells by inducing apoptosis and inhibiting proliferation *via* cell cycle arrest.^3^

**Fig. 1:**
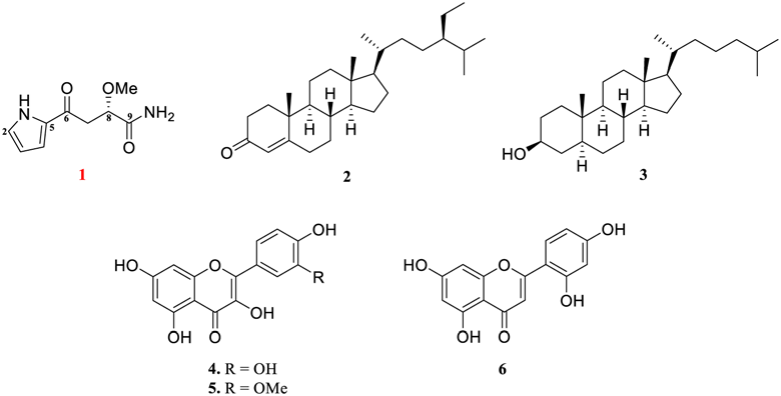
Chemical structure of compounds 1–6 isolated from the roots of *N. nucifera* Gaertn.

In our ongoing effort to discover intriguing natural products from herbs, the roots of *N. nucifera* Gaertn. were chosen for chemical investigation. The result of isolation is one novel pyrrole terpenoid hydrid, named lotusin A (1), and five known including sterol analogs, cholestanol (2) and stigmast-4-en-3-one (3) and flavonoid analogs, quercetin (4), isorhamnetin (5), norartocarpetin (6) were characterized (Fig. 1). Compounds 1 and 2 inhibited the level of NO production, while all the compounds showed growth inhibition against six bacterial strains, namely, *S. aureus*, *B. subtilis*, *M. luteus*, *S. typhimurium*, *E. coli*, and *K. pneumoniae*, as well as inhibition of α-glucosidase. Molecular docking simulation, *in silico* ADMET and toxicity predictions are also reported. The isolation, structural elucidation, and biological activities (*in vitro* and *in silico*) of the isolated compounds are presented herein.

## Results and discussion

**Fig. 2:**
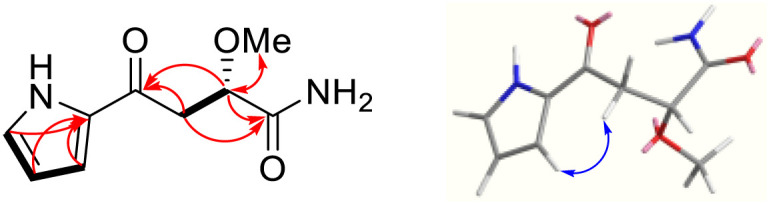
Key COSY, HMBC, and ROESY correlations of 1.

Lotusin A was isolated as a whitish amorphous powder. The molecular formula of C_9_H_12_N_2_O_3_ was determined by HR-ESIMS with *m*/*z* 219.0747 [M + Na]^+^ (calcd for C_9_H_12_N_2_O_3_Na^+^, 219.0746), implying five degrees of unsaturation. The UV absorption was displayed at a maximum of 292.2 nm, while the IR absorption featured bonds at 3324 (NH) and 1657 (carbonyl) cm^−1^. The ^1^H NMR spectrum of 1 (Table 1) indicated the characteristic proton signals with chemical shifts at *δ*_H_ 7.08, 7.02, and 6.24, and typical small coupling constants (3.6, 2.4, and 1.2 Hz). The combination of ^13^C NMR and HSQC data suggested that these protons were linked to carbon signals at *δ*_C_ 127.0, 118.7, and 111.2 for C-2, 3, and 4, respectively, which was confirmed by the literature reports,^4–10^ leading to the conclusion that 1 contained a mono-substituted pyrrole ring and accounted for three of the five degrees of unsaturation. Subsequently, a set of oxygenated methine signals at *δ*_H_ 4.23 (dd, *J* = 9.0, 3.6) and *δ*_C_ 79.7 (CH), a pair of un-equivalent methylene protons at *δ*_H_ 3.21 (dd, *J* = 15.6, 9.0 Hz), 3.05 (dd, *J* = 15.6, 3.6 Hz) and *δ*_C_ 42.3 (CH_2_), a methoxy at *δ*_H_ 3.38 (s) and *δ*_C_ 59.0 (OCH_3_), together with ketone and carbonyl groups at *δ*_C_ 188.0 (C-6) and 177.8 (C-9), respectively, were reminiscent of the (α)-methoxysuccinic acid moiety containing a terpenoid core and satisfied the fourth and fifth degree of unsaturation. The connection of the terpenoid core and the pyrrole ring through the single bond C-5–C-6 was confirmed by the correlation of H-4 and H-7 in the ROESY data (Fig. 2). This indicated that a pyrrole terpenoid hybrid was established. The amide functionality (C-9) was confirmed by the odd-mass value in the scaffold, containing two nitrogen heterocyclic atoms. The configuration of 1 was well defined by a comparison of specific optical rotation with the reported values. The optical rotation value of 1 ([*α*]25_D_ −16.6 (c 5.0, acetone)) was in good agreement with those of (*S*)-α-methoxysuccinic acid ([*α*]_D_ −25.0 (c 5.0, acetone))^11,12^ and (*S*)-malic acid ([*α*]_D_ −25.8 (c 5.5, pyridine)),^13^ whereas (*R*)-malic acid ([*α*]_D_ +25.5 (c 5.5, pyridine))^13^ had an opposite value (Fig. S.7). Thus, the structure of 1 was conclusively determined.

**Table 1:** ^1^H NMR and ^13^C NMR data of compound 1^a^

Position	1 (ppm)	
	*δ*_H_ (*J* in Hz)	*δ*_C_
2	7.08, dd (2.4, 1.2)	127.0
3	6.24, dd (3.6, 2.4)	111.2
4	7.02, dd (3.6, 1.2)	118.7
5	—	133.2
6	—	188.0
7a	3.21, dd (15.6, 9.0)	42.3
7b	3.05, dd (15.6, 3.6)	
8	4.23, dd (9.0, 3.6)	79.7
9	—	177.8
8-OCH_3_	3.38, s	59.0

a ^1^H NMR (600 MHz) and ^13^C NMR (150 MHz) measured in methanol-*d*_4_.

The pyrrole scaffold contained a privileged *N*-heterocyclic atom, reflecting in the structures of many natural products and the variety of biological activities. Pyrrole derivatives have been demonstrated by a wide range of therapeutic applications and distribution into a variety of commercial drugs: prodigiosin-anticancer, atorvastatin-lipid-lowering, remdesivir-antiviral, indomethacin-NSAID, and nargenicin-antibacterial.^14^ Regarding natural pyrrole derivatives, the herbal medicine source exhibited a modest amount, whereas they were frequently found in secondary metabolites from marine organisms.^15^ For the former, 3-substituted pyrrole alkaloids such as solsodomine A and B were reported from fresh berries of *Solanum sodomaeum* L.,^16^ while other derivatives with the 1, 2, 5-trisubstitution accounted for somewhat crowd and were isolated from *Quararibea funebris*, *Pisum satiuum* and *Grifola frondosa*.^17–21^ Combined pyrrole and terpenoid structures have been reported solely from *Streptomyces* metabolites, totaling nineteen derivatives to the best of our knowledge.^4–10^ However, all previously reported compounds are monosubstituted pyrrole derivatives, in which the pyrrole ring is linked to sesquiterpenoid (C_15_) units at the C-2 position. In contrast, compound 1 represents a distinctive example within this structural class, featuring a C-2 substituent that is a butanoic acid (C_4_) derivative instead of the typical sesquiterpenoid moiety. Therefore, compound 1 is considered as a unique example of a pyrrole terpenoid motif possessing a C-2 substituent, which is a butanoic acid (4C) derivative. This is the first report on the occurrence of one pyrrole terpenoid type from a plant (Table S1).

Furthermore, two known sterol and three known flavonoid analogs were identified as cholestanol (2),^22^ stigmast-4-en-3-one (3)^23^ and quercetin (4),^24^ isorhamnetin (5)^25^ and norartocarpetin (6)^26^ by spectroscopic analysis and comparison with those reported in the literature.

It is based on combined biological activities of reported pyrrole sesquiterpenes and available screening models in our lab, including those regarding anti-inflammatory activities, antibacterial activities, and inhibition of α-glucosidase. Compounds 1 and 2 were assayed in terms of the inhibition of NO production, pro-inflammation, and the minimal inhibitory concentration (MIC) against Gram-positive (*S. aureus*, *B. subtilis*, and *M. luteus*) and Gram-negative (*S. typhimurium*, *E. coli*, and *K. pneumoniae*) bacteria. The result indicated that 1 displayed a moderate effect on the inhibition of NO production (IC_50_ = 21.5 µM), whereas 2 did not show any activity. The inhibition of NO production was reported for compounds 3–6 in a different manner of the assay; thus, we did not repeat the same motif of nitrile oxide production.^27–30^ To further evaluate the biological potential of compounds 1–6, we examined their antibacterial activities through preliminary screening at a concentration of 10 µM. The assays were conducted against three Gram-positive bacterial strains (*Staphylococcus aureus*, *Bacillus subtilis*, and *Micrococcus luteus*) and three Gram-negative strains (*Salmonella typhimurium*, *Escherichia coli*, and *Klebsiella pneumoniae*). This comparative screening aimed to determine whether any of these compounds could inhibit bacterial growth under identical conditions by conducting further experiments. However, as summarized in Table 2, none of the tested compounds exhibited inhibitory effects against any of the six bacterial strains. In diabetes model, 1 also displayed good inhibition of α-glucosidase with an IC_50_ value of 18.2 µM.

**Table 2:** Bioactivities of isolated compounds^a^

Bioactivities		Compounds						Positive control
		1	2	3	4	5	6	
NO production (IC_50_, µM)		21.5 ± 0.3	N.A.	—	—	—	—	Celastrol 1.2 ± 0.1
α-Glucosidase (IC_50_, µM)		18.2 ± 0.17	N.A.	N.A.	N.A.	N.A.	N.A.	Acarbose 197.0 ± 3.8
MIC (10.0 µM)		—	—	—	—	—	—	Kanamycin
Gram-positive	*Staphylococcus aureus*	N.A.	N.A.	N.A.	N.A.	N.A.	N.A.	A.
	*Bacillus subtilis*	N.A.	N.A.	N.A.	N.A.	N.A.	N.A.	A.
	*Micrococcus luteus*	N.A.	N.A.	N.A.	N.A.	N.A.	N.A.	A.
Gram-negative	*Salmonella typhimurium*	N.A.	N.A.	N.A.	N.A.	N.A.	N.A.	A.
	*Escherichia coli*	N.A.	N.A.	N.A.	N.A.	N.A.	N.A.	A.
	*Klebsiella pneumoniae*	N.A.	N.A.	N.A.	N.A.	N.A.	N.A.	A.

a Data are an average of at least three tests. N.A.: not active; A.: active; “—”: did not test.

**Fig. 3:**
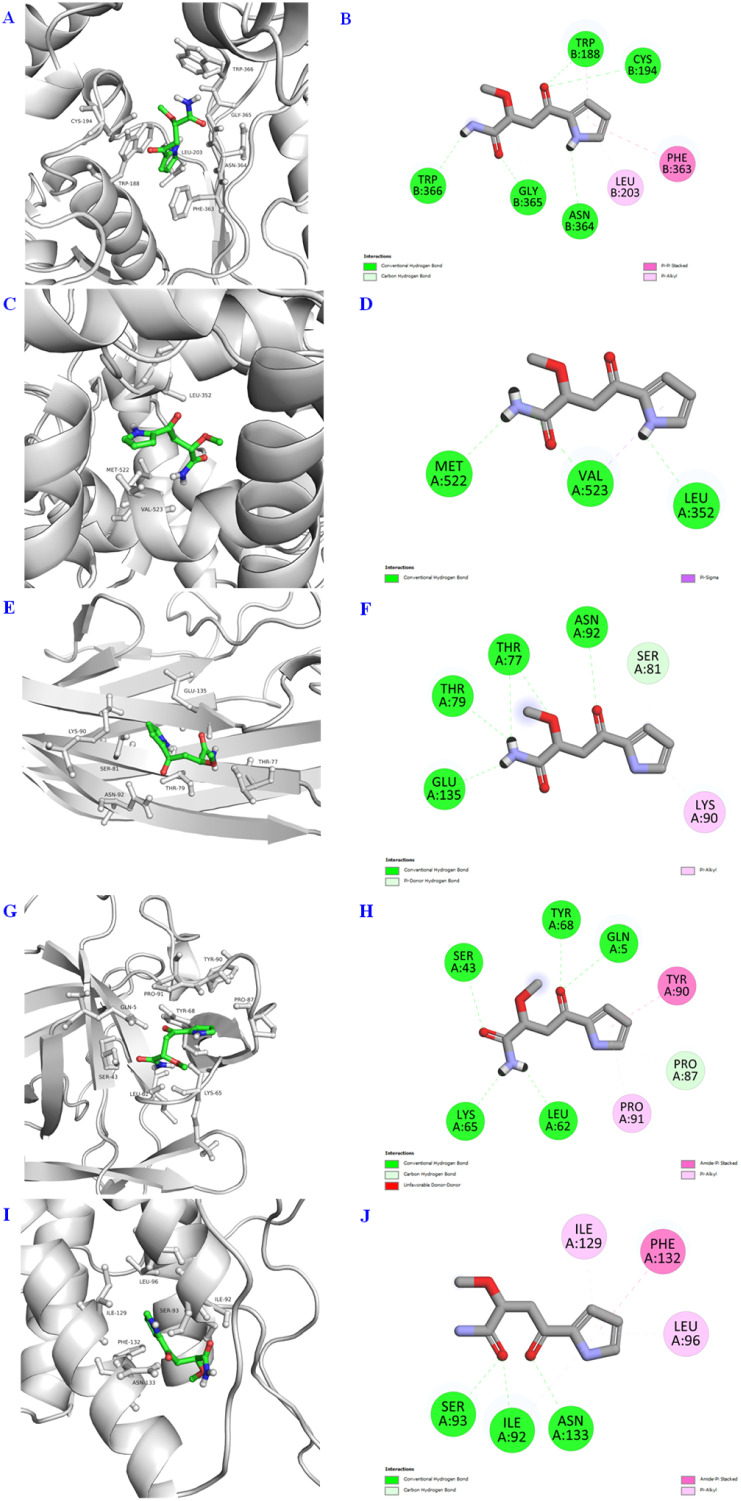
3D docking poses and 2D interaction diagrams of pro-inflammatory cytokines including iNOS (A and B), COX-2 (C and D), TNF-α (E and F), IL-1β (G and H), and IL-6 (I and J) with compound 1.

**Fig. 4:**
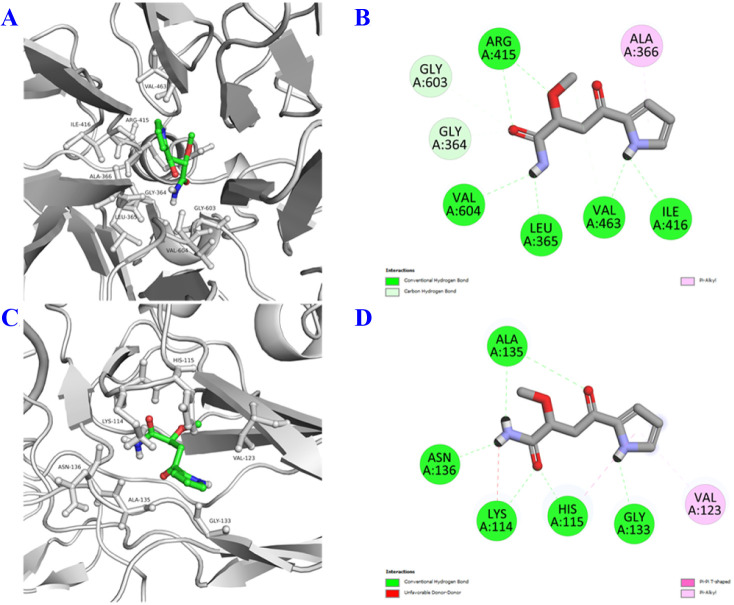
3D docking poses and 2D interaction diagrams of transcription factors including Nrf2 (A and B) and NF-κB (C and D) with compound 1.

**Fig. 5:**
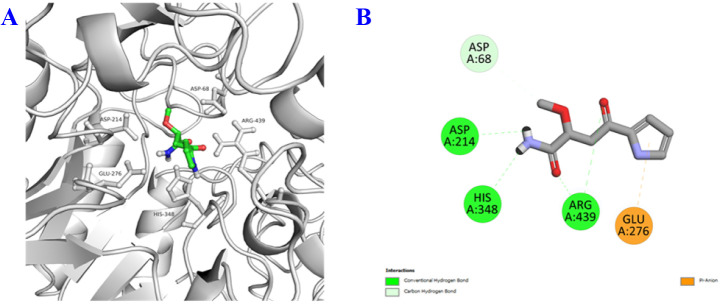
3D docking poses and 2D interaction diagrams of α-glucosidase (A and B) with compound 1.

In the *in vitro* model, compound 1 exhibited inhibition of NO production as well as α-glucosidase; besides, it is a unique structure from plant natural products. Therefore, we wish to study *in silico* prediction to provide a comprehensive relative insight. The binding interactions of 1 are summarized in Fig. 3–5 and Table 3. The results showed that 1 has good interaction with both pro-inflammatory cytokines and transcription factors, as well as diabetes enzyme with binding energies ranging from −5.0 to −6.6 kcal mol^−1^. These computational results aligned quite well with the *in vitro* data, presented in detail in Table 2. Notably, all binding affinities referred specially, and accounted mostly, to hydrogen bond interactions, but other interactions were also present to some extent. In correlation of structural functionality and studied proteins, it is indicated that (i) the amide group exhibited good binding affinities to all predicted proteins by two-five H-bond interactions, (ii) the ketone functionality showed its affinities, making to second important position, (iii) the amine bonding of the pyrrole unit was also not less important in the same docking model, interacting with most of the protein crystal structures. Generally, although the chemical structure of 1 is quite small (C_9_H_12_N_2_O_3_), compared to others, it contained most functionalities enough to make interactions of docking simulation (Table 3).

**Table 3:** Molecular docking interactions of compound 1 with pro-inflammatory cytokines (iNOS, COX-2, TNF-α, IL-1β, and IL-6), transcription factors (Nrf2 and NF-κB), and α-glucosidase

Cytokines/enzyme	Proteins (PDB ID)	Binding energy (kcal mol^−1^)	Interactions and bond length (Å)
iNOS	1M8D	−6.4	Hbond: Trp188 (3.23), Cys194 (3.41), Gly365 (3.04), Asn364 (2.84), Trp366 (2.53)
			Carbon Hbond: Gly365 (3.48)
			π–π Stacked: Phe363 (4.72)
COX-2	6COX	−6.4	Hbond: Met522 (2.38), Val523 (2.49), Leu352 (2.58)
			π-sigma: Val523 (3.59)
TNF-α	2TNF	−5.0	Hbond: Glu135 (2.14), Thr79 (2.58), Thr77 (2.47), Thr77 (2.93), Asn92 (3.15)
			π-donor Hbond: Ser81 (3.84)
IL-1β	8I1B	−5.8	Hbond: Leu62 (2.19), Lys65 (2.21), Ser43 (2.91), Tyr68 (2.71), Gln5 (2.82)
			Carbon Hbond: Pro87 (3.38)
			Amide-π stacked: Tyr90 (3.87)
IL-6	2L3Y	−6.1	Hbond: Ser93 (2.06), Ile92 (2.16), Asn133 (2.11)
			Amide-π stacked: Phe132 (4.79)
			π-alkyl: Ile92 (4.72), Ile129 (4.72), Leu96 (4.93)
Nrf2	3WN7	−6.6	Hbond: Ile416 (2.87), Val463 (2.51), Leu365 (2.47), Val604 (2.64), Arg415 (3.01), Arg415 (3.23)
			Carbon Hbond: Gly364 (3.47), Gly603 (3.48), Val463 (3.76)
			π-alkyl: Ala (4.29)
NF-κB	1NFK	−5.5	Hbond: Gly133 (2.42), His115 (1.92), Lys114 (2.47), Asn136 (2.31), Ala135 (2.52), Ala135 (2.61)
			Unfavorable donor–donor: Lys114 (2.69)
α-Glucosidase	MAL32	−6.4	Hbond: His348 (2.41), Asp214 (2.66), Arg439 (3.07), Arg439 (3.07)
			Carbon Hbond: Asp68 (3.60)
			π-anion: Glu276 (4.51)

During the inflammatory processes, large amounts of pro-inflammatory mediator, nitric oxide (NO), are generated by the inducible NO synthase (iNOS) and COX-2.^31^ iNOS protein is expressed response in a variety of pro-inflammatory cytokines^32,33^ whereas COX-2 protein is only detectable in certain types of tissues and is induced transiently by pro-inflammatory cytokines.^34,35^ Besides iNOS and COX-2 proteins, pro-inflammatory cytokines such as TNF-α, IL-1β, and IL-6 were reported as the biological functions of NF-κB. TNF-α plays a key role in the induced and maintained inflammation due to autoimmune reactions by T cell activation by upregulating other pro-inflammatory cytokines and endothelial adhesion molecules such as intercellular and vascular cell adhesion molecule 1 (IAM1 and VCAM1), increasing the recruitment of leukocytes to inflammatory sites.^36^ Similarly, IL-1β protein is one of the most important inflammatory cytokines secreted by macrophages, and is LPS induction in macrophages. During inflammation, increases in the release of IL-1β lead to cell or tissue damage,^37,38^ and thus, reduction in IL-1β release from macrophages may retard inflammatory responses. Additionally, the production of IL-6 is induced by factors of TNF-α and IL-1β. A proinflammatory cytokine of IL-6 acts as an endogenous pyrogen in addition to its multiple effects on the immune system. These cytokines are regulated by the signal transduction pathway of NF-κB activation.^39^ It is assumed that Nrf2 and NF-κB signalling pathways cooperate to maintain the physiological homeostasis of cellular redox status and to regulate the cellular response to stress and inflammation. However, the molecular mechanisms underlying this functional interaction appear to be specific to cell type and tissue, and are still under elucidation.^40^ Although *in silico* models cannot absolutely replace experiments of *in vitro* and *in vivo* assay, based on the above-mentioned data, it is estimated that compound 1 showed anti-inflammatory inhibition through the Nrf2 and NF-κB signalling pathways. They may also provide valuable approach and overview insights to estimate the signalling pathways of anti-inflammatory mechanism for further *in vitro* and *in vivo* assays and allow scientists to target the potential effects and direct the early stages of research pipeline, optimizing time and resources.

As a result, the *in silico* ADMET prediction of 1 using the ADMET lab 3.0 web tool is shown in detail in Table 4. Our work adhered to Lipinski's Rule of Five, suggesting that they are likely to be bioavailable orally. The results also indicated a high probability of gastrointestinal absorption for 1, contributing to their favourable bioavailability. Cytochrome P450 (CYP) is a group of enzymes including CYP1A2, CYP2C19, CYP2C9, CYP2D6, and CYP3A4 predominantly found in the liver and intestines, responsible for metabolizing most drugs *via* oxidation processes. However, compound 1 did not inhibit these CYP enzymes, reducing the drug efficacy or even causing toxicity.

**Table 4:** *In silico* ADME and toxicity profiles of 1

ADME profiles		Toxicity profiles	
Molecular weight	196.21	Acute LD_50_ (mg kg^−1^)	1357.41
No. H-bond acceptor	5	Carcinogenesis	Safe
No. H-bond donor	3	Liver injury I (DILI)	Safe
No. Rotatable bonds	4	Liver injury II	Toxic
TPSA (Å^2^)	85.18	Micronucleus	Toxic
log *P*	0.05	hERG blockers	Safe
log *S*	−1.14	Androgen receptor	Safe
Gastrointestinal absorption	High	Androgen receptor-LBD	Safe
log *k*_p_ (cm s^−1^)	−2.16	Estrogen receptor	Safe
CYP1A2 inhibitor	No	Estrogen receptor-LBD	Safe
CYP2C19 inhibitor	No	Glucocorticoid receptor	Safe
CYP2C9 inhibitor	No	Thyroid receptor	Safe
CYP2D6 inhibitor	No	—	—
CYP3A4 inhibitor	No	—	—
Lipinski violations	0 violation	—	—

Next, the toxicity profiles of compound 1 were predicted by applying the Deep-PK computational tool. Compound 1 exhibited average toxicity with an LD_50_ value of 1357.41 and a value greater than 1000 mg kg^−1^ suggested that 1 has a relatively low risk of acute toxicity. Moreover, the predicted result makes a consideration relating to a potential to bind with the liver injury II and micronucleus. It is indicated that this interaction may contribute to liver trauma, which can run the gamut of minor lacerations or capsular hematomas and formation in a cell when chromosomes or chromosome fragments are not incorporated into the main nucleus during cell division. The prediction models for each endpoint and values are established based on different model types and training datasets; thus, those data may have discrepant final results. Additionally, 1 displayed significant interactions with main receptors including the carcinogenesis, liver injury I (DILI), hERG blockers, androgen receptor, androgen receptor-LBD, estrogen receptor, estrogen receptor-LBD, glucocorticoid receptor, and thyroid receptor. These predicted data of *in silico* toxicity were the most commonly investigated and reported in toxicology, suggesting that 1 may hold promise for further study.

## Conclusions

A chemical profile investigation of *N. nucifera* roots led to the identification of six compounds, namely, one novel pyrrole terpenoid hybrid, named lotusin A (1), together with two known sterols (2 and 3) and three known flavonoids (4–6). Among them, compound 6 was isolated for the first time from this species. Compound 1 showed the inhibition of NO production (IC_50_ = 21.5 µM), while others had no antibacterial effect at a preliminary concentration of 10 µM. Compound 1 also displayed inhibition of α-glucosidase (IC_50_ = 18.2 µM). In addition, molecular docking studies were performed to explore the possible interactions between compound 1 and key pro-inflammatory cytokines, transcription factors and α-glucosidase. The docking models revealed favorable binding affinities between 1 and the target proteins, supporting their potential biological relevance. Complementary *in silico* toxicity predictions indicated a low likelihood of adverse effects, further reinforcing their safety profile. Taken together, these computational findings suggest that compound 1 may serve as a promising candidate for further investigation, particularly through *in vitro* and *in vivo* experiments focused on its preliminary anti-inflammatory and antidiabetic potential.

## Experimental

### General experimental procedures

The NMR spectra were recorded using a Varian Unity Inova 600 MHz spectrometer with tetramethylsilane (TMS) as an internal standard, and the chemical shifts were recorded in *δ* values (ppm). High-resolution mass spectra were recorded using an X500R QTOF system (SCIEX, Framingham, MA). UV spectra were recorded using a Thermo spectrometer. IR spectra were recorded using a JASCO FT/IR-4100 spectrometer. Silica gel (Merck, 63–200 µm particle size), RP-18 (Merck, 75 µm particle size), and Sephadex LH-20 were used for column chromatography. TLC was performed using Merck silica gel 60 F_254_ and RP-18 F_254_ plates. Preparative HPLC was performed using a water system with a UV detector 2996 and a YMC-Triart C18 column (10 × 250 mm, 5 µm particle size, YMC Co., Ltd, Japan). Compounds were visualized with aqueous 10% H_2_SO_4_ after heating for 3–5 min.

### Plant material

Lotus roots were dug at Thap Muoi district, Dong Thap Province, Vietnam (10°31′44.4″N 105°43′15.9″E). The faced description of the sample closed resemble to depiction of professor Do Tat Loi.^41^ The general morphological features are similar to those of *Nelumbo nucifera* Gaertn. The voucher specimens (registry no. MNP002) are deposited at the Department of Organic and Medicinal Chemistry, Faculty of Pharmacy, University of Health Sciences, Vietnam National University Ho Chi Minh City (UHS-VNU).

### Extraction and isolation

The Lotus roots of *N. nucifera* (dried, 6.8 kg) were extracted absolutely three times (24 h × 20 L) with industrial methanol at room temperature. After the solvent was removed under reduced pressure, the residue was suspended in warm water and then fractionated in order of *n*-hexane, CH_2_Cl_2_ (MC), and EtOAc (EA) each 10 L, successively. The chromatography of the MC-soluble fraction (54.0 g) was performed using a silica gel column (80 × 12 cm, 63–00 µM particle size, Merck) with a stepwise gradient of Hx–EA (10 : 1 to 0 : 1, each 2 L) to yield 13 fractions (Fr. MC1-Fr. MC13) according to their TLC profiles. Fraction MC3 (5.4 g) was successively eluted to a silica gel column chromatograph (60 × 6.5 cm) eluting with Hx–EA (10 : 1, each 2 L) to yield 8 fractions (Fr. MC3.1 to MC3.8). Fraction MC3.6 (600 mg) was continuously eluted using a YMC RP-18 column with MeOH–H_2_O (5:1 to 1:0, each 0.5 L) to give 6 fractions (Fr. MC3.6.1–MC3.6.6). Fraction MC3.6.2 (235.0 mg) was repeated using a Sephadex silica gel column with a solvent system of MeOH : H_2_O (9 : 1, each 0.5 L), which yielded 5 fractions. Fraction MC3.6.2.2 (87.5 mg) was further purified over a semi-preparative Waters HPLC system with an isocratic solvent system of 30% MeOH in H_2_O + 0.1% formic acid (flow rate = 2 mL min^−1^) over 60 min, UV detection at 210 and 254 nm as an eluent to yield 1 (1.4 mg), 4 (2.4 mg), 5 (3.7 mg). In the same manner, compounds 2 (3.8 mg), 3 (4.4 mg), and 6 (3.9 mg) were also isolated from faction MC3.1.

### Lotusin A (1)

Whitish amorphous powder; UV *λ*_max_ (MeOH) (log *ε*): 292.2 (1.0) nm; IR (ATR) *ν*_max_: 3324, 2939, 1657, 1448, 1416 and 1021 cm^−1^; ^1^H and ^13^C NMR (MeOH-*d*_4_) data, see Table 1 and SI; HR-ESIMS *m*/*z* 219.0747 [M + Na]^+^ (calcd. for C_9_H_12_N_2_O_3_Na^+^, 219.0746).

### MTT assay for cell viability^42^

The MTT assay was performed using a slightly modified version of a previously reported method.^42^ The cell viability was determined based on 24 h of continuous exposure of RAW 264.7 cell to compounds 1 and 2 by a colorimetric assay. Briefly, 1 × 10^4^ cells per well were treated for 24 h with positive control or compounds for cell viability. The viability of the macrophages treated with vehicle (0.5% DMSO) only was defined as 100%. The survival of macrophage was calculated using the following formula: viable cell number (%) = OD_570_ (treated cell culture)/OD_570_ (vehicle control) × 100.

### Determination of NO production^42^

The production of nitric oxide (NO) was quantified by measuring the NO levels in cell culture supernatants using a previously method. In brief, RAW264.7 cells (ATCC, Rockville, MD, USA, 1 × 10^5^ cells per well) were treated with 1 µg mL^−1^ of LPS for 24 h, with and without the test compounds (1–100 µM). After incubation, the supernatant (100 µL) was mixed with 100 µL of Griess reagent. The viability of the remaining cells was assessed using an MTT-based colorimetric assay as described above.

### MIC measurement^43^

The antibacterial activities of compounds 1–6 were evaluated in 96-well plates using a modified broth microdilution method previously reported by our group. The MIC of the isolated compounds was determined against *Staphylococcus aureus*, *Bacillus subtilis*, Micrococcus luteus, *Salmonella typhimurium*, *Escherichia coli*, and *Klebsiella pneumonia*. Bacterial strains were cultured on TSB (Tryptic Soy Broth) agar plates and incubated at 37 °C for 24 h. A single colony was transferred to Muller–Hinton broth (MHB), continuously incubated and harvested after 24 h at 37 °C and centrifuged at 250 rpm. The suspensions were adjusted to 0.5 McFarland standard, diluted to achieve a final concentration of approximately 5 × 10^5^ CFU mL^−1^ and added to each well. All compounds were dissolved in DMSO and dispensed into 96-well plates at a starting concentration of 10 µM. Kanamycin was used as a positive control.

### α-Glucosidase inhibitory assay^44^

The α-glucosidase inhibitory assay was performed using a somewhat modified method of Kurihara *et al.*^44^ For every reaction, 50 mL of 1.5 mM *p*-nitrophenyl-α-d-glucopyranoside, 50 mL of 0.1 U mL^−1^, α-glucosidase in 0.01 M phosphate buffer (pH 7.0), 625 mL of sample solution (various concentrations of compound 1 with 5, 10, 25, 50 µM) were mixed. The mixture was kept at 37 °C for 30 minutes and added with 0.1 M Na_2_CO_3_. Mixture after reaction will be measured at a wavelength of 401 nm. The IC_50_ values represented the inhibitor concentration that suppressed 50% of enzyme activity. Acarbose was used as the positive control. All experiments were performed in triplicate. The percentage inhibition was calculated using the following equation: α-glucosidase inhibition (%) = (*A*_c_ − *A*_t_)/*A*_c_ × 100, where *A*_c_ is the absorbance of control (α-glucosidase, without sample) and *A*_t_ is the absorbance of test samples (α-glucosidase, with sample). The experimental results were processed using the GraphPad Prism 8 software.

### Molecular docking methods

Molecular docking was conducted to predict protein–ligand binding affinities and identify potential ligand-binding sites,^45^ providing valuable insights into the biological properties of the studied systems.^46^ Docking was performed using AutoDock Vina.^47^ The protein crystal structures of *Mus musculus*-derived proteins, including inducible nitric oxide synthase (iNOS, PDB ID: 1M8D),^48^ cyclooxygenase-2 (COX-2, PDB ID: 6COX),^49^ tumor necrosis factor-alpha (TNF-α, PDB ID: 2TNF),^50^ interleukin-1 beta (IL-1β, PDB ID: 8I1B),^51^ interleukin-6 (IL-6, PDB ID: 2L3Y),^52^ Kelch-like ECH-associated protein 1-nuclear factor erythroid 2-related factor 2 (Keap1-Nrf2, PDB ID: 3WN7),^53^ and nuclear factor kappa B (NF-κB, PDB ID: 1NFK),^54^ were retrieved from the RCSB Protein Data Bank (https://www.rcsb.org/). As the crystallographic structure of *Saccharomyces cerevisiae* α-glucosidase was unavailable, the 3D structure, designated AF-P38158-F1 (MAL32), was retrieved from the AlphaFold Protein Structure Database (https://alphafold.ebi.ac.uk/entry/P38158). This AlphaFold-derived model was validated as the most reliable representation of *S. cerevisiae* α-glucosidase in the absence of an experimentally determined crystal structure.^55^ Prior to molecular docking, each protein crystal structure was preprocessed by removing water molecules, heteroatoms, and co-factors using standard protocols to obtain a clean protein model. For docking studies involving iNOS (1M8D), COX-2 (6COX), and MAL32, a grid box with dimensions of 23 Å × 23 Å × 23 Å was defined to encompass the region of interest. For the remaining proteins, blind docking was performed to explore possible ligand-binding interactions. Molecular docking simulations were conducted, and the resulting binding poses and interactions were visualized using PyMOL (version 1.3.1., the PyMoL molecular graphics system, Chrödinger LLC. 2010) and Discovery Studio Visualizer (v25.1.0.24284, BIOVIA, Dassault Systèmes, 2025).

### *In silico* ADME and toxicity profiles

*In silico* ADMET predictions^56^ of compound 1 were performed using the ADMET lab 3.0 web tool (https://admetlab3.scbdd.com/, accessed 21st July 2025). The SMILES format of 1 was used to compute the key physicochemical properties, and ChemDraw 21.0.0 was used for evaluation. Compound 1 was also analyzed for their pharmacokinetic profiles, including gastrointestinal absorption, Caco-2 permeability, plasma protein binding and interaction with cytochrome P450. The toxicity predictions^57^ of compounds were made using the DEEP-PK web tool (https://biosig.lab.uq.edu.au/deeppk/prediction, accessed 21st July 2025), which included classification into toxicity categories and estimation of the median lethal dose (LD_50_) and interaction between compound and biological targets critical to key physiological processes, offering valuable insights into the safety and potential risks of 1.

## Author contributions

Conceptualization: Le Viet Ha Tran, Huu Canh Vo; isolation and identification: To Hoang Long, Quoc Tuan Le, Minh Canh Nguyen, Duc Trung Le, Thanh-Tung Phan; bioassay: Vinh Han La, Linh Tran; computational studies: Le-Thuy-Thuy-Trang Hoang; writing – original draft preparation: Tran Dang Linh Nguyen, Ngo Thi Thuy Duong; writing – review and editing: Quang Ton Thai, Huynh Nguyen Khanh Tran; supervision: Khac-Minh Thai; project administration: Minh-Tri Le. All authors have read and agreed to the published version of the manuscript.

## Conflicts of interest

The authors declare no competing financial interest.

## Supplementary Material

RA-015-D5RA05354D-s001

## Data Availability

The data that support the findings of this study are available in the supplementary information (SI). Supplementary information: spectral data of novel compound (1). See DOI: https://doi.org/10.1039/d5ra05354d.
