# Supplementary material for: Lotusin A: a novel pyrrole terpenoid hydrid from lotus roots (Nelumbo nucifera Gaertn.)
Source: RSC Adv. 2025 Dec 15;15(58):50103–10. doi: 10.1039/d5ra05354d (PMC12705226; doi:10.1039/d5ra05354d)
Supplement: RA-015-D5RA05354D-s001 [file RA-015-D5RA05354D-s001.pdf]

Supplementary Information (SI) for RSC Advances.
This journal is © The Royal Society of Chemistry 2025

Supplementary Material

Lotusin A, a novel pyrrole terpenoid hydrid from the Lotus roots (*Nelumbo nucifera* Gaertn.)

Le Viet Ha Tran[a,c,e], Huu Canh Vo[b,c,e], To Hoang Long[b,c,e], Vinh Han La[c,e], Quoc Tuan Le[c,e], Minh Canh Nguyen[c,e], Duc Trung Le[c,e], Tran Dang Linh Nguyen[c,e], Thanh-Tung Phan[b,e], Ngo Thi Thuy Duong[b,e], Quang Ton That[b,e], Linh Tran[,d,e], Minh-Tri Le[c,e], Khac-Minh Thai[c,d,e], Le-Thuy-Thuy-Trang Hoang[f,g], Huynh Nguyen Khanh Tran[c,d,e*]

[a] Faculty of Traditional Medicine, University of Medicine and Pharmacy at Ho Chi Minh City

[b] Faculty of Chemistry, University of Science, Ho Chi Minh City 70000, Vietnam

[c] Faculty of Pharmacy, University of Health Sciences, Ho Chi Minh City, Vietnam

[d] Research Center for Discovery and Development of Healthcare Products Vietnam National University Ho Chi Minh City, Ho Chi Minh City, Vietnam

[e] Vietnam National University, Ho Chi Minh City 70000, Vietnam

[f] Laboratory of Advanced Materials Chemistry, Institute for Advanced Study in Technology, Ton Duc Thang University, Ho Chi Minh City, Vietnam

[g] Faculty of Applied Sciences, Ton Duc Thang University, Ho Chi Minh City, Vietnam

*Corresponding author

E-mail: thnkhanh@uhsvnu.edu.vn / hnktran2404@gmail.com

Tel: +84939775593

ORCID: 0000-0003-4472-0280

ABSTRACT

Nelumbo nucifera Gaertn. has been used as traditional medicines and food. Up to the present, many unknown constituents are still being discovered. Most of the research about secondary metabolites and their pharmacological properties focus on alkaloid derivatives. In this study, one novel pyrrole terpenoid hydrid, lotusin A (**1**), and five reported substances, cholestanol (**2**), stigmast-4-en-3-one (**3**), quercetin (**4**), isohamnetin (**5**), and norartocarpetin (**6**) were isolated from methanol extract from the roots of *N. nucifera* Gaertn.. The structures (**1**–**6)** were identified *via* spectroscopic analyses of NMR (1D and 2D), HR-ESIMS, and comparisons with previous literatures. Compound **1**, a pyrrole terpenoid hydrid, is a unique isolation from plant natural products. **1** and **2** tested with inhibitory activity of NO production whereas all compounds absorbed growth inhibition assay of six bacterial strains. The results indicated that **1** moderately inhibited NO production with an IC_{50} value of 21.5 µM but no inhibition of microbial growth at concentration of 10 µM. Besides, compound of **1** also exhibited the good inhibition of *α*-glucosidase (IC_{50} = 18.2 µM), in contrast, the result did not satisfy to others. From molecular docking result, **1** has good interaction to with both of pro-inflammatory cytokines (iNOS, COX-2, TNF-α, IL-1β, and IL-6); transcription factors (Nrf2 and NF-κB), diabetes enzyme with binding energies of −6.4, −6.4, −5.0, −5.8, −6.1 kcal/mol; −6.1, −6.6 kcal/mol; and −6.4 kcal/mol, respectively, resulted that **1** is the promising candidate for anti-inflammatory and *α* -glucosidase inhibition. Moreover, *in silico* ADMET and toxicity predictions indicated that **1** had favourable safety and pharmacokinetic profiles.

Keywords: Lotus root, *Nelumbo nucifera* Gaertn., pyrrole, NO production, anti-inflammatory, *α*-glucosidase

List of Figures

Fig.S.1. HR-ESIMS spectrum of compound **1**

Fig.S.2. ^{1}H NMR spectrum of compound **1** in methanol-d_4 at 600 MHz

Fig.S.3. ^{13}C NMR spectrum of compound **1** in methanol-d_4 at 150 MHz

Fig.S.4. HSQC spectrum of compound **1** in methanol-d_4

Fig.S.5. COSY spectrum of compound **1** in methanol-d_4

Fig.S.6. HMBC spectrum of compound **1** in methanol-d_4

Fig.S.7. ROESY spectrum of compound **1** in methanol-d_4

Fig.S.8. Comparison of specific optical rotation of **1** and reported values

List of Figures

Table S1. Systematic comparison of isolated pyrrole terpenoids from natural source

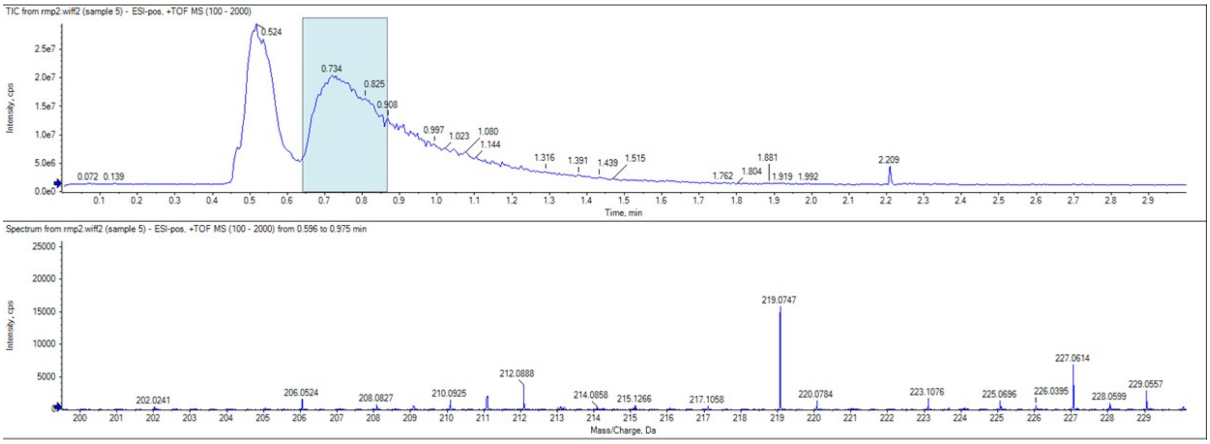

Fig.S.1. HR-ESIMS spectrum of compound **1**

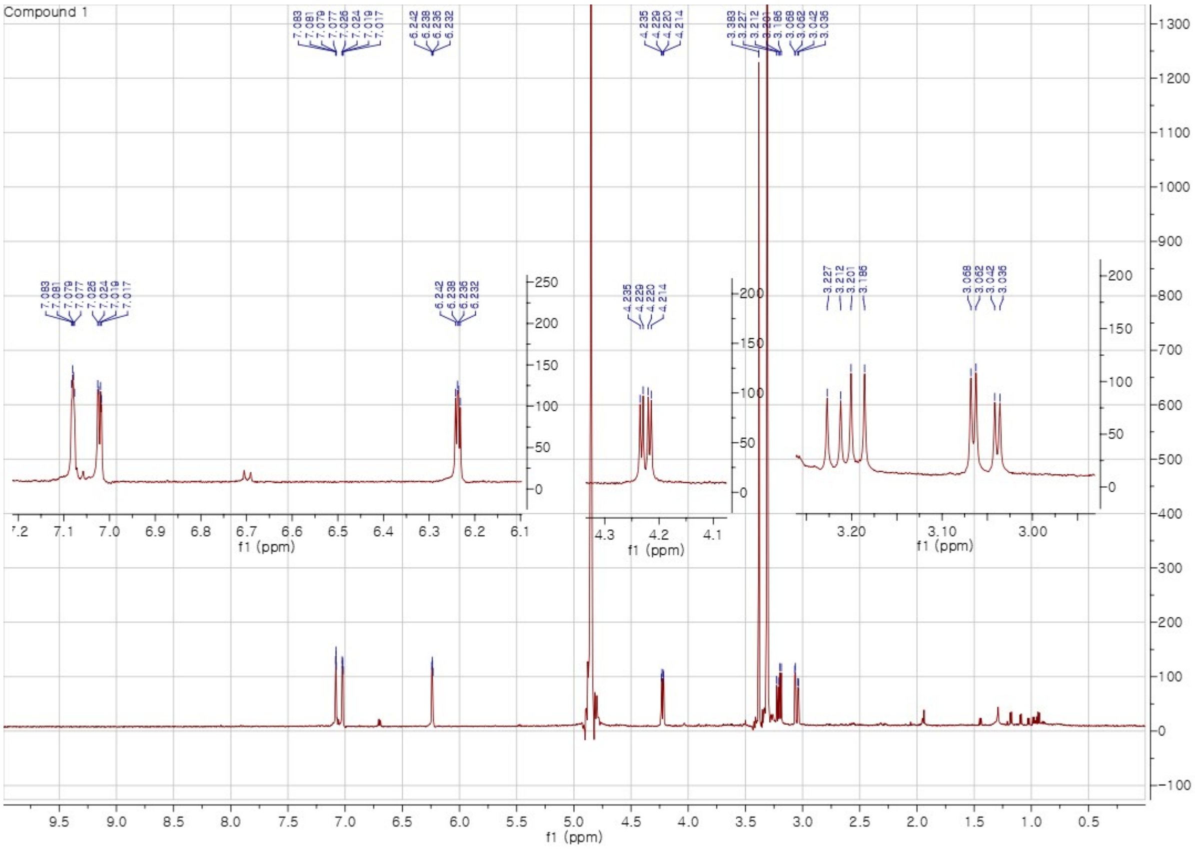

Fig.S.2. ^{1}H NMR spectrum of compound **1** in methanol-d_4 at 600 MHz

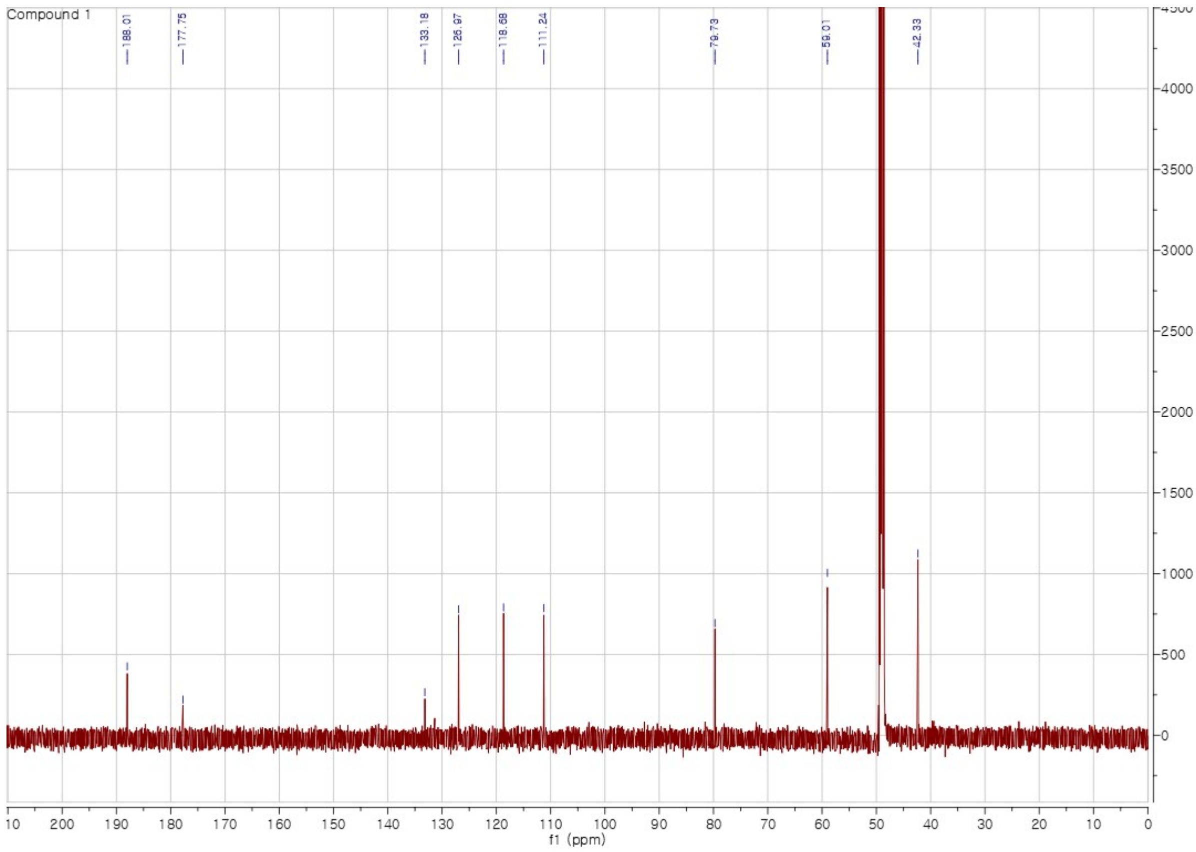

Fig.S.3. ^{13}C NMR spectrum of compound **1** in methanol-d_4 at 150 MHz

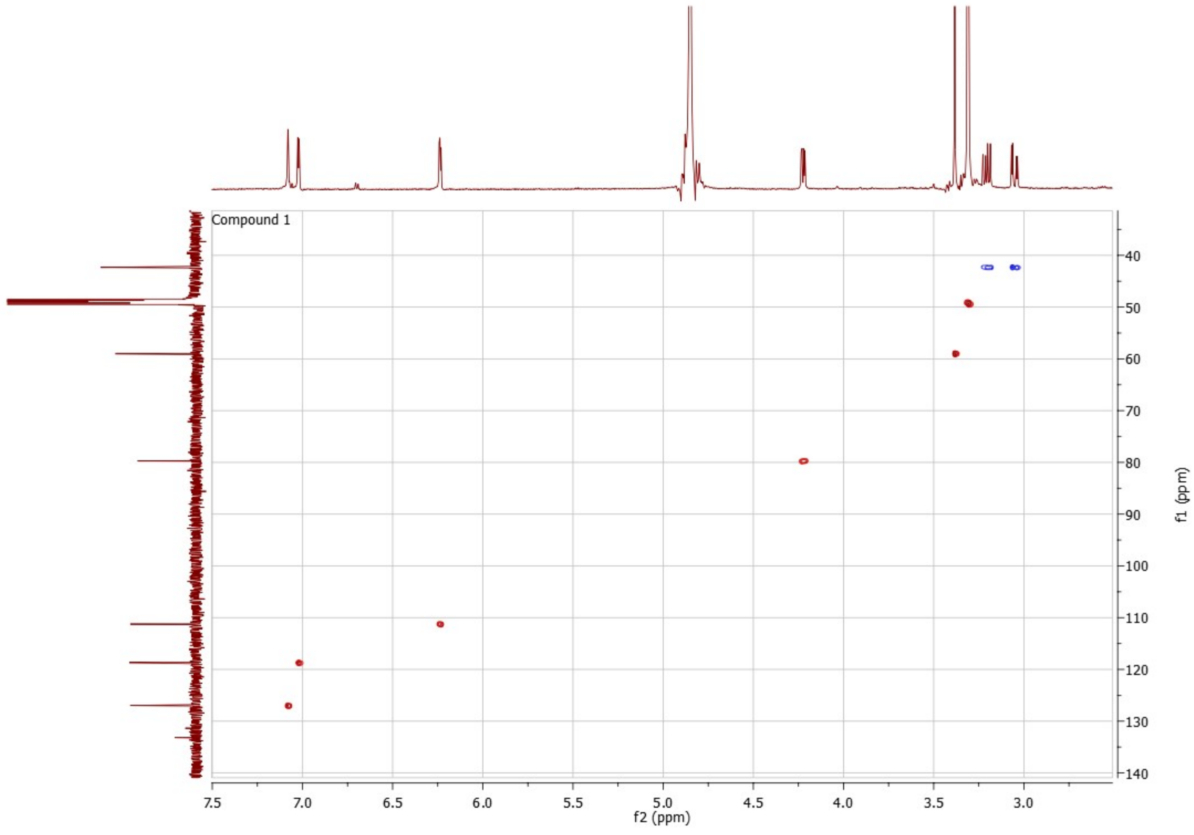

Fig.S.4. HSQC spectrum of compound **1** in methanol-d_4

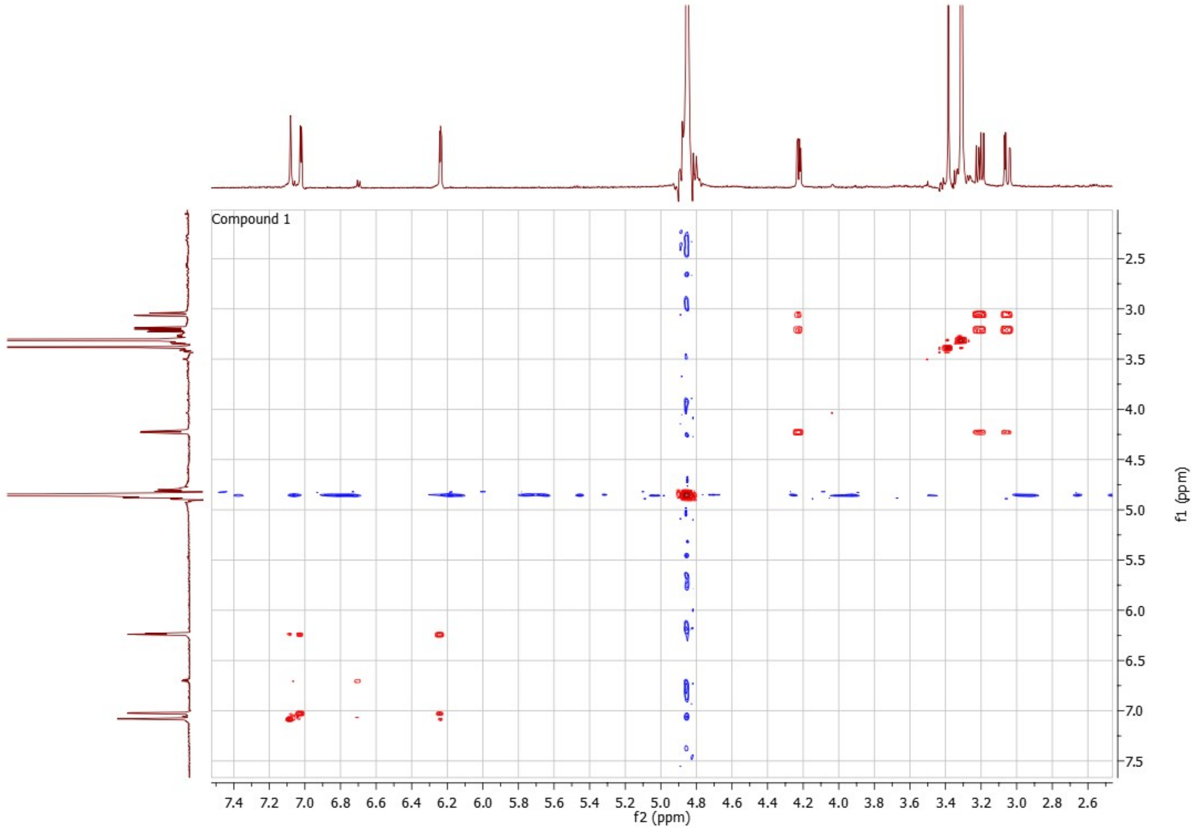

Fig.S.5. COSY spectrum of compound **1** in methanol-d_4

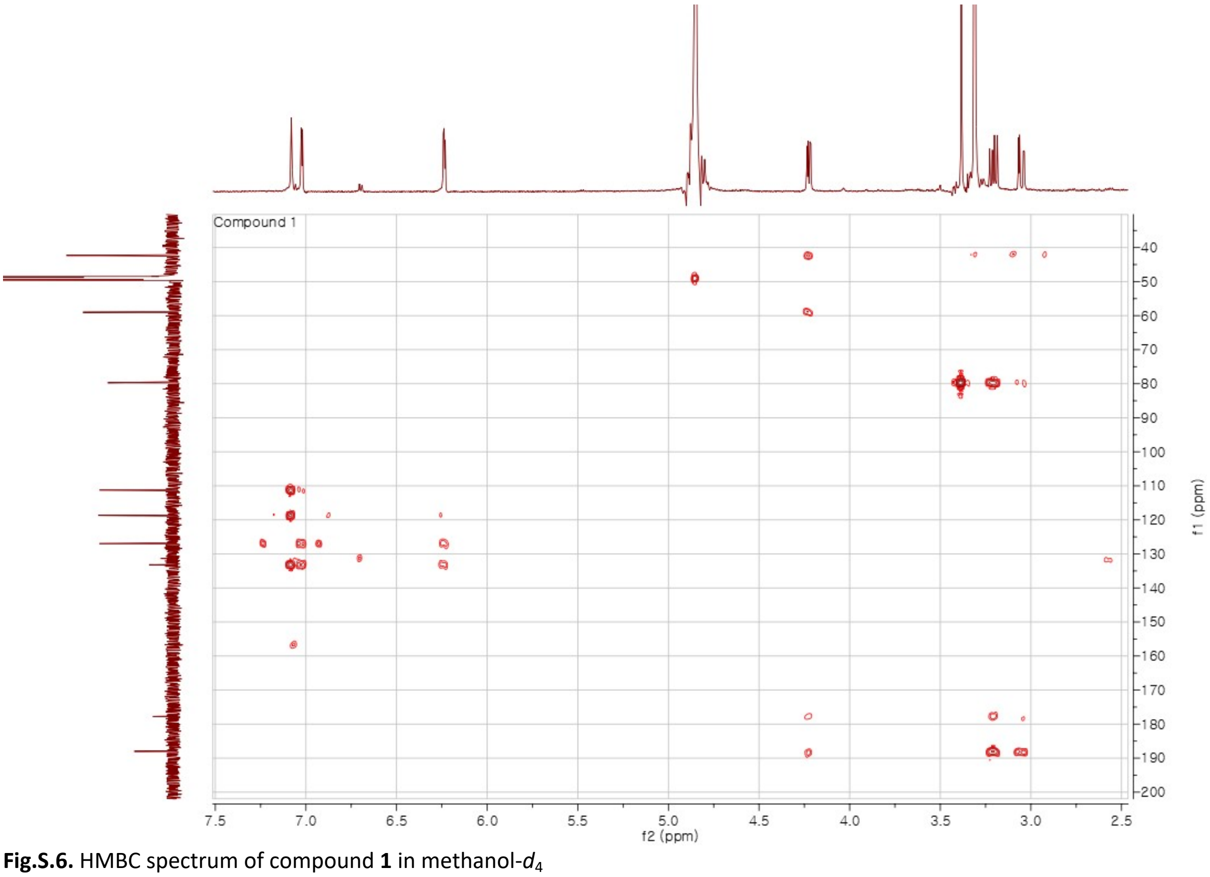

Fig.S.6. HMBC spectrum of compound **1** in methanol-d_4

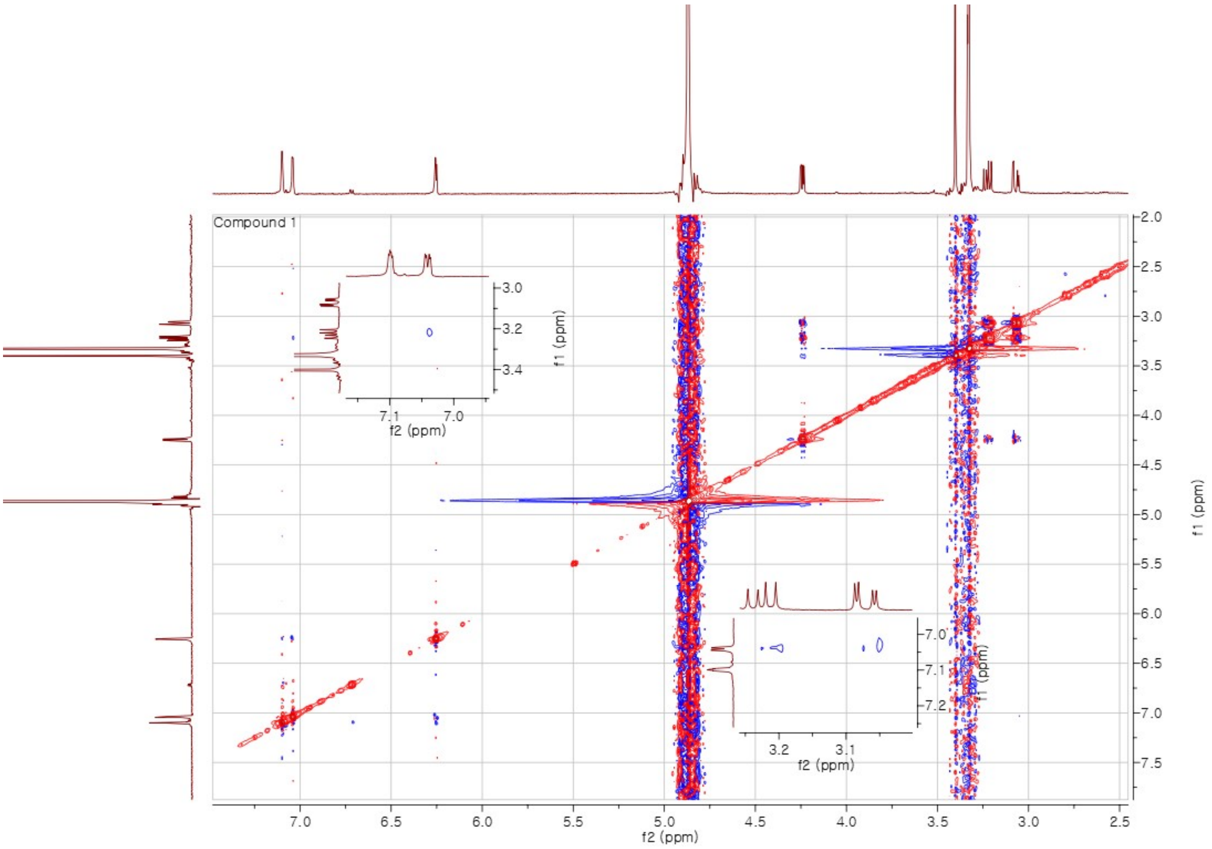

Fig.S.7. ROESY spectrum of compound **1** in methanol-d_4

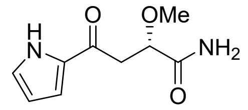

1 (*S*)

$[\alpha]^{26}_{D} = -16.6$ (*c* 5.0, acetone)

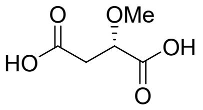

(*S*)-*α*-methoxysuccinic acid

$[\alpha]^{27}_{D} = -25.0$ (*c* 5.0, acetone)

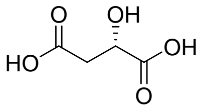

(*S*)-malic acid

$[\alpha]_{D} = -25.8$ (*c* 5.5, pyridine)

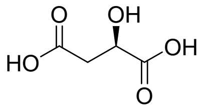

(*R*)-malic acid

$[\alpha]_{D} = +25.5$ (*c* 5.5, pyridine)

Fig.S.8. Comparison of specific optical rotation of **1** and reported values

Table S1. Systematic comparison of isolated pyrrole terpenoids from natural source

No.	Name	Structure	Source	Reference [4-10]
1	glaciapyrrole A	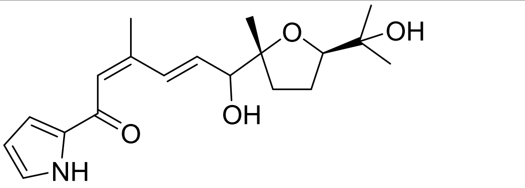	*Streptomyces* sp. (NPS008187)	Journal of natural products 68.5 (2005): 780-783.
2	glaciapyrrole B	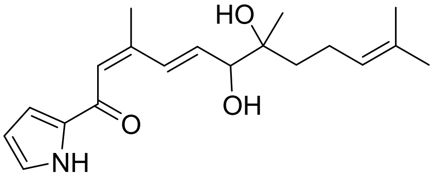		
3	glaciapyrrole C	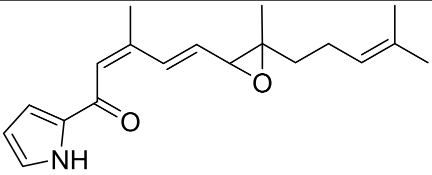		
4	pyrrolostatin	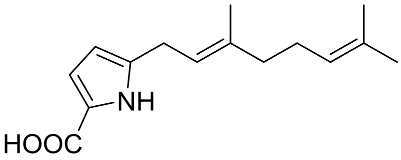		
5	heronapyrrole A	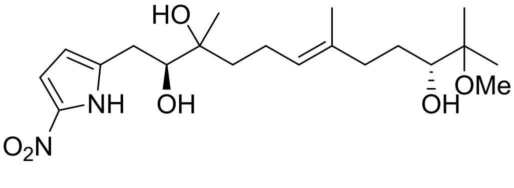	*Streptomyces* sp. (CMB-M0423)	Organic letters 12.22 (2010): 5158-5161.
6	heronapyrrole B	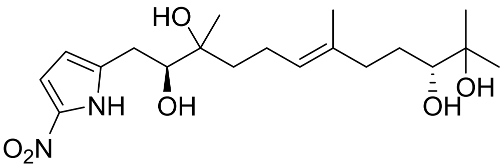		
7	heronapyrrole C	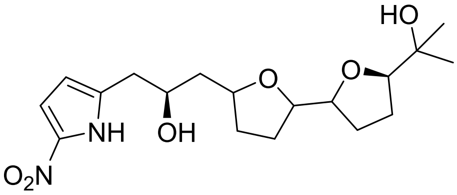		
8	-	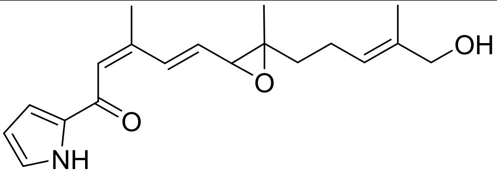	*Streptomyces* sp.	Magnetic Resonance in Chemistry 52.1-2 (2014): 57-59.

9	-	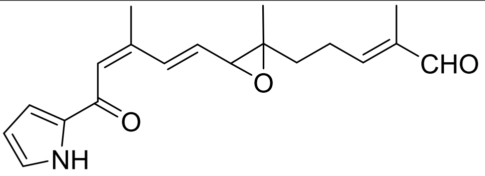		
10	-	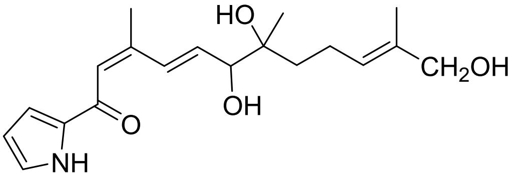	*Streptomyces* sp.	Natural Product Communications 9.4 (2014): 1934578X1400900402
11	-	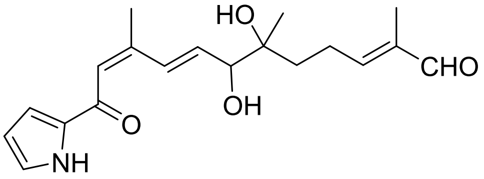	*Streptomyces* sp. Hd7–21	The Journal of Antibiotics 67.5 (2014): 415-417
12	(2*Z*,4*E*,9*E*)-6,7,11-trihydroxy-3,7,11-trimethyl-1-(1*H*-pyrrol-2-yl)dodeca-2,4,9-trien-1-one	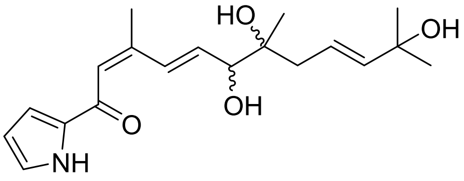	*Streptomyces* sp. Hd7–21	Chemistry & Biodiversity 12.1 (2015): 153-156
13	(2*Z*,4*E*)-5-{3-[(2*E*)-4-hydroxy-4-methylpent-2-en-1-yl]-3-methyloxiran-2-yl}-3-methyl-1-(1*H*-pyrrol-2-yl)penta-2,4-dien-1-one	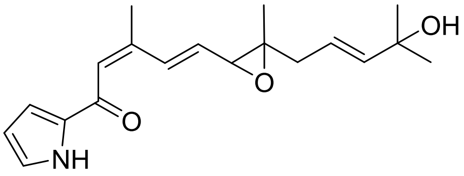		
14	strepyrrolin A	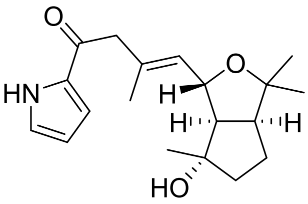	*Streptomyces* sp. KIB 015	Organic Chemistry Frontiers 10.4 (2023): 880-889.

15	strepyrrolin B	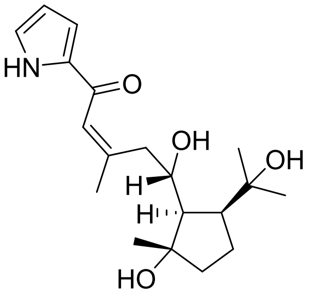		
16	strepyrrolin C	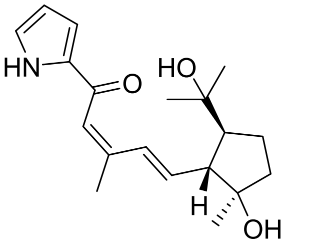		
17	strepyrrolin D	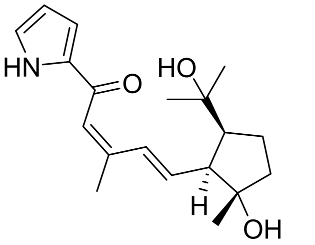		
18	strepyrrolin E	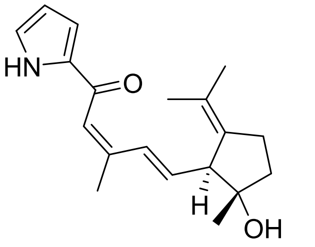		
19	-	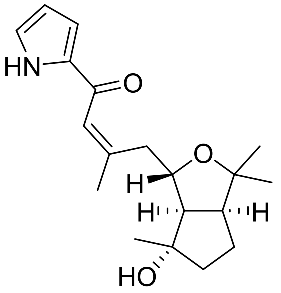		
20	Compound 1	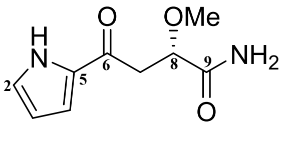	***Plant:*** *Nelumbo nucifera* Gaertn.	-

References

4. V. R. Macherla, J. Liu, C. Bellows, S. Teisan, B. Nicholson, K. S. Lam, B. C. Potts, *J. Nat. Prod.*, 2005, **68**, 780-

783.

5. R. Raju, A. M. Piggott, L. X. Barrientos Diaz, Z. Khalil, R. J. Capon, *Org. Lett.*, 2010, **12**, 5158-5161.
6. D. Z. Liu and B. W. Liang, *Magn. Reson. Chem.*, 2014, **52**, 57-59.
7. D.-Z. Liu, B.-W. Liang, *Nat. Prod. Commun.*, 2014, **9**, 451-452.
8. D.-Z. Liu, B.-W. Liang, *J. Antibiot.* 2014, **67**, 415-417.
9. D. Z. Liu, B. W. Liang, X. F. Li, *Chem. Biodivers.*, 2015, **12**, 153-156.
10. L. Wang, Z. Zhou, J.-P. Huang, L. Zhang, X. Tuo, Z. Yu, S.-X. Huang, Y. Liu, J. Yang, *Org. Chem. Front.s*, 2023, **10**, 880-889.
